# Ultrasound-Guided Comparison of Psoas Compartment Block and Supra-Inguinal Fascia Iliaca Compartment Block for Pain Management in Pediatric Developmental Dysplasia of Hip Surgeries

**DOI:** 10.3389/fped.2021.801409

**Published:** 2022-02-02

**Authors:** Junjun Quan, Shujun Yang, Yuchao Chen, Kai Chen, Siyuan Yu

**Affiliations:** ^1^Department of Anesthesiology, Ministry of Education Key Laboratory of Child Development and Disorders, Chongqing Key Laboratory of Pediatrics, National Clinical Research Center for Child Health and Disorders, Children's Hospital of Chongqing Medical University, Chongqing, China; ^2^Department of Orthopedics, Children's Hospital of Chongqing Medical University, Chongqing, China

**Keywords:** psoas compartment block, supra-inguinal fascia iliaca block, developmental dysplasia of the hip, perioperative pain management, sufentanil

## Abstract

**Background:**

The aim of this study was to compare psoas compartment block (PCB) and supra-inguinal fascia iliaca compartment block (SFIB) in terms of pain management and the need for additional systemic analgesia in the perioperative phase of developmental dysplasia of the hip (DDH).

**Materials and Methods:**

Sixty pediatric patients were randomized into the PCB group and the SFIB group. The Numeric Rating Scale (NRS) pain scores were used to assess postoperative pain during the initial 24 h after extubation. Sufentanil consumption, patient-controlled analgesia (PCA) demands, and complications were also recorded.

**Results:**

The NRS pain scores were significantly lower in the PCB group than in the SFIB group at 0, 4, 8, 12, and 24 h after extubation (all *P* < 0.01). Postoperatively, 13.8% of patients in the PCB cohort received additional administration of sufentanil, in contrast to 63.3% of the SFIB cohort (*P* < 0.01). In the PCB group, 0 (0-0) mcg/kg sufentanil was administered, while in the SFIB group 0.1 (0-0.2) mcg/kg (*P* < 0.01). In addition, the PCB group had fewer PCA demands than the SFIB group within the initial 24 h (*P* < 0.01). It took less operating time to achieve SFIB as compared to PCB (*P* < 0.01). No adverse events related to two techniques were recorded.

**Conclusions:**

PCB provided a better perioperative pain management in pediatric patients with the DDH surgeries compared to SFIB. It also reduced the need for supplementary systemic analgesia.

## Introduction

Perioperative pain management of patients undergoing surgeries for developmental dysplasia of the hip (DDH) is a challenging issue for anesthesiologists. The pediatric patients with DDH at late stage and concomitant hip pathology or other related abnormities maybe only receive orthopedic surgeries such as acetabuloplasty combined with femoral osteotomy (1). This surgical approach involves a large area of tissues innervated mostly by the lumbosacral plexus (2), therefore, for older children with unilateral hip and femoral surgeries, regional block has become a promising and advantageous analgesic technique in perioperative pain management (3). In practical work, the most efficacious form of regional anesthesia for unilateral hip and femoral surgeries has yet to be determined, especially in pediatric patients (3, 4).

Psoas compartment block (PCB) is a traditional regional block technique through injecting local anesthetic into the iliopsoas muscle compartment. And the local anesthetic diffuses proximally to surround and anesthetize the lumbar plexus to relieve perioperative pain. To date, ultrasound-guided PCB combined with sciatic nerve block has been successfully applied to hip and femur surgeries in adult patients (5, 6), which reveals great perioperative analgesic effects. Based on a growing body of work on local anesthetics, several studies have recently demonstrated that local anesthetics are able to spread in dorsal and proximal directions toward the lumbar plexus, which promotes the development of supra-inguinal fascia iliaca compartment block (SFIB) (7, 8). The spread potentially improves the success of lateral femoral cutaneous nerve block when compared with the inferior-inguinal fascia iliaca compartment block and has already shown its strength in adult hip and femur surgeries (7, 8). Soft tissue connections such as nerve sheath and aponeurosis are looser in children compared to adults, which favors the diffusion of local anesthetics and produces easier block of the distal nerve fibers. A few studies have recommended SFIB as a promising alternative for perioperative analgesia in hip and femur surgeries due to its simplicity (9), but its efficiency has been still questioned (10). Nevertheless, there is a dearth of studies available assessing the application of PCB and SFIB in pediatric DDH surgeries at present.

This study was, therefore, aimed to compare the perioperative analgesic efficiency and the advantages of PCB and SFIB. The primary outcomes were Numeric Rating Scale (NRS) pain scores, additional opioid consumption, and patient-controlled analgesia (PCA) demands. The secondary outcomes included operating time to achieve block and the adverse events. We hypothesized that there would be no difference in pain scores between patients that received PCB versus SFIB in children with the DDH surgeries.

## Materials and Methods

This study was approved by the Institutional Review Board of Children's Hospital of Chongqing Medical University, Chongqing, P.R China. The trial was registered at the Chinese Clinical Trial Registry (ChiCTR1900027277) on November 7, 2019 prior to patient enrollment. Written informed consents were obtained from all subjects.

A total of 60 subjects were enrolled and randomized in a 1:1 ratio into two groups, the PCB group and the SFIB group. The participants were 6-14 years old. All included subjects were admitted to the hospital between November 2019 to January 2020, with a diagnosis of DDH and an American Society of Anesthesiologists (ASA) physical status of I to II, and all were scheduled to undergo acetabuloplasty and femoral osteotomy in one limb. Patients who had contraindications to nerve block, refused to the items or did not enter the post-anesthesia care unit (PACU) for receiving nerve block were excluded.

All subjects after entering operation room were connected to GE Healthcare CARESCAPE ™ Monitor B650 to monitor electrocardiograph, non-invasive blood pressure, heart rate, oxygen saturation. Three mg/kg of propofol, 0.2 μg/kg of sufentanil and 0.1 mg/kg midazolam were used for sedation. Oxygen at the concentration of 100% was administrated under the mask in all patients after satisfactory sedation. The two groups received nerve block with local anesthetics. In the SFIB group, the patients received ultrasound-guided SFIB as described by Hebbard et al. (11), while the patients in the PCB group received PCB under the guidance of ultrasound and neurostimulator as described by Chayen et al. (12). In addition, both groups underwent subgluteal sciatic nerve block. Needles used for nerve block were the same size (22G ^*^ 80 mm). Subsequently, tracheal intubation was performed after intravenous anesthesia with administration of 3 mg/kg of propofol, 0.6 μg/kg of sufentanil and 0.2 mg/kg of cis-atracurium. After intubation, the inspiratory oxygen concentration was maintained at 35%, whereas the exhaled carbon dioxide at 35-40 mmHg. 2-3% of sevoflurane and 0.2-0.3 mcg/kg/min of remifentanil were used to maintain anesthesia. Norepinephrine was administered when the patients had unstable blood pressure due to massive hemorrhage (indication for blood transfusion: hemoglobin level < 7 g/dl). Blood was collected intraoperatively using Sorin Xtra^®^ autotransfusion system for cell salvaging in both groups.

Local anesthetic 0.25% ropivacaine (AstraZenca AB, Sweden) was used at 1 ml/kg for SFIB and PCB. The dose ratio between the local anesthetics used in SFIB/PCB and in sciatic nerve block was at 8:2 (the dose of local anesthetic for SFIB or PCB was not more than 35 ml). All blocks were performed by the same experienced attending anesthesiologist. Two resident anesthesiologists blinded to all nerve block procedures provided subsequent anesthesia management and recorded the relevant data. The patients were transferred to PACU after surgery. The intravenous infusion channel was connected to a PCA pump when the patients were awake and extubated. All patients were extubated after fulfilling extubation criteria with responding to verbal commands, achieving adequate spontaneous ventilation and full recovery from muscle relaxation with a train of four ratio > 0.9 on neuromuscular transmission monitoring. If the NRS pain score of a patient was above 3 in PACU, an additional dose of sufentanil (0.1 μg/kg) was intravenously administered immediately. The patients were transferred to the ward when meeting the PACU discharge criteria.

Clinical data including medical history (age at admission, gender, weight, and height), allogeneic blood transfusion, nerve block-related complications, operating time to achieve block SFIB or PCB, operation time, PACU duration, and hospital stay after surgery were collected. Postoperative sufentanil consumption was recorded after the patients were transferred to PACU. NRS pain scores (no pain: 0; mild pain: 1-3; moderate pain: 4-6; severe pain: 7-10; worst pain: 10) were documented at 0, 4, 8, 12, and 24 h after extubation. PCA administration within the initial 24 h was also noted.

## Statistical Analysis

The mean NRS pain score at 24 h of 1.14 ± 0.66 in the PCB group and 2.5 ± 0.65 in the SFIB group, with a two-sided type I error of 0.05 and a power of 0.90, 60 subjects in total (30 patients in each group) were found to be enough for statistical significance. Sample size calculation was performed using PASS (version 15.0). The normality of data distribution was detected with the use of the Shapiro–Wilk test. Continuous data were presented as mean ± standard deviation or median (interquartile range [IQR]) and analyzed by Student's *t*-test or Mann-Whitney *U*-test. Categorical data were presented as number (%) and analyzed by Chi-square test. Statistical analysis was performed using SPSS (version 23.0, IBM Corporation, Armonk, NY, USA). *P*-value < 0.05 was considered statistically significant.

## Results

**Figure 1:**
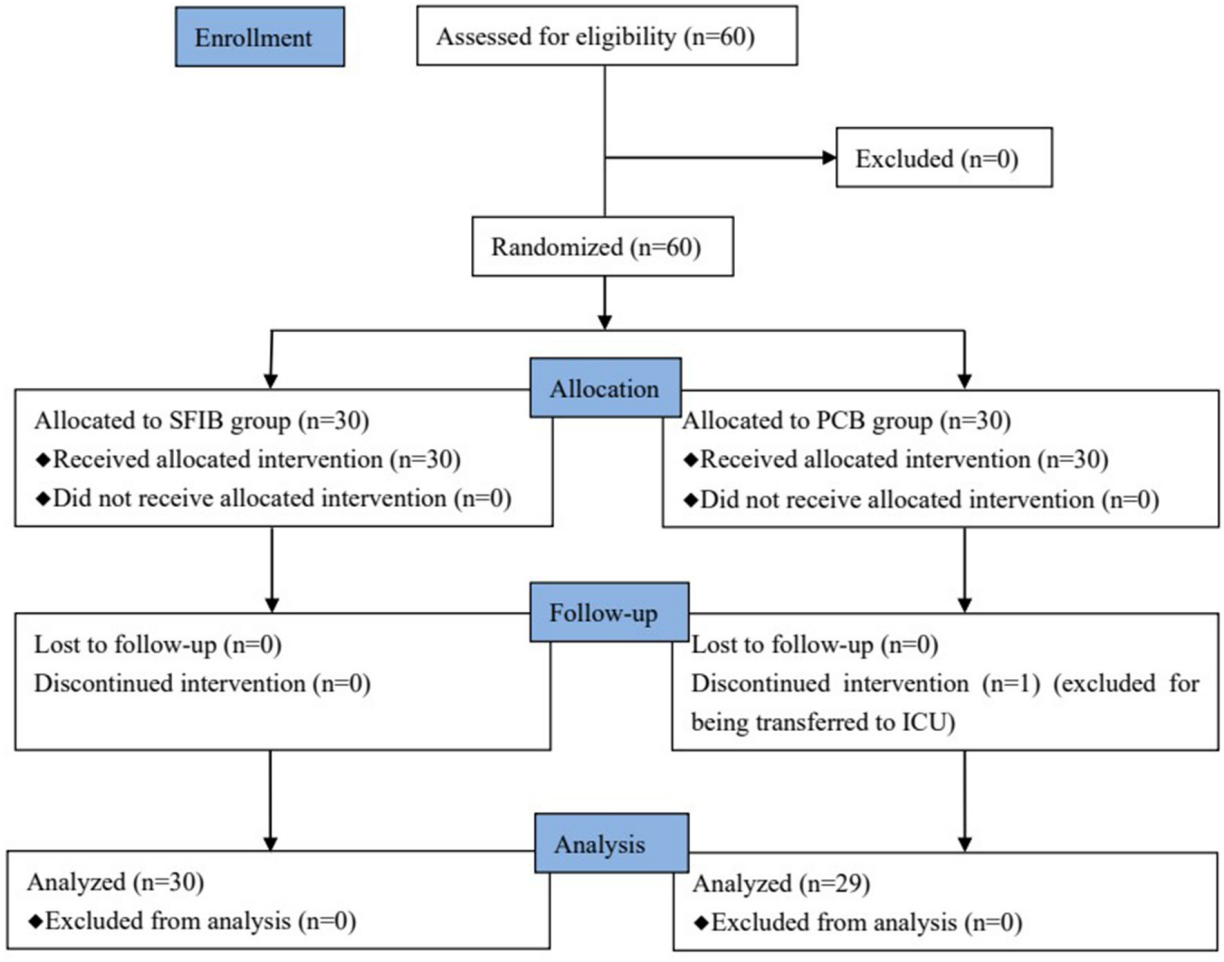
Consort flow diagram for screening and enrollment. PCB, psoas compartment block; SFIB, supra-inguinal fascia iliac compartment block.

After consent, sixty pediatric patients were randomized in a 1:1 ratio to receive either PCB or SFIB, as shown in Figure 1. One patient in the PCB group was transferred to Intensive Care Unit postoperatively and excluded from this study. There were no statistically significant differences between the groups in age (*P* = 0.79), sex (*P* = 0.07), and body mass index (*P* = 0.29) as demonstrated in Table 1. The average ages were 8.69 ± 2.41 and 8.93 ± 2.24 years old in the PCB group and the SFIB group, respectively. There were 19 females and 10 males in the PCB group, along with 18 female and 12 male patients in the SFIB group (Table 1).

**Table 1:** Demographic characteristics.

**Variables**	**PCB (*n* = 29)**	**SFIB (*n* = 30)**	***P***
Sex (Famale/Male)	19/10	18/12	0.79
Age (years)	8.69 ± 2.41	8.93 ± 2.24	0.07
BMI (kg/m^2^)	19.36 ± 0.62	19.54 ± 0.69	0.29
Blood transfusion (%)	5 (17.2%)	4 (13.3%)	0.73
Nerve block-related complications (%)	0	0	/
Time to achieve block (min)	5 (5-5.75)	3 (2.5-3.5)	<0.01
Time to surgery (h)	4.5 (4.5-5)	4.5 (4-5)	0.05
PACU duration (min)	30 (25-35)	30 (25-30)	0.59
Hospital stay after surgery (day)	8 (7-10)	8 (7-9)	0.71

*Continuous data are presented as mean ± standard deviation or median [IQR] and analyzed by Student's t-test or Mann-Whitney U-test. Categorical data are presented as number (%) and analyzed by Chi-square test. BMI, body mass index; PACU, post-anesthesia care unit; PCB, psoas compartment block; SFIB, supra-inguinal fascia iliaca compartment block*.

The operating time to achieve the nerve block in the PCB group was significantly longer than in the SFIB group (5 [5-5.75] vs. 3 [2.5-3.5] min, *P* < 0.01). There were no significant differences between the groups in surgical operating time (*P* = 0.05), PACU duration (*P* = 0.59), hospital stay after surgery (*P* = 0.71), and incidence of allogeneic blood transfusion (*P* = 0.73, Table 1). There were no patients administrated with intraoperative vasopressors, and no cases reported as to nerve block-related complications.

The NRS pain scores were significantly lower in the PCB group as compared with the SFIB group at 0, 4, 8, 12, and 24 h (2 [1-2] vs. 4 [2-5], 2 [1.5-2] vs. 4 [4-5], 2 [2-2.5] vs. 4 [3.75-4], 1 [1-2] vs. 3 [3-4], 1 [0.5-1] vs. 3 [2-3]; all *P* < 0.01) after extubation, as shown in Table 2. The percentage of patients with NRS pain score ≤ 3 in the PCB group was higher than that in the SFIB group during the initial 24 h after extubation (all *P* < 0.01, Table 3). Nearly 100% of the patients in the PCB group had mild pain (NRS pain score: 1-3) from 0 to 24 h, while the most patients in the SFIB group had moderate pain (NRS pain score: 4-6). And the SFIB cohort reached the same pain score range only at 24 h after extubation, taking the PCB cohort as a reference.

**Table 2:** NRS pain score after extubation.

**Time (h)**	**PCB (*n* = 29)**	**SFIB (*n* = 30)**	***P***
0	2 (1-2)	4 (2-5)	<0.01
4	2 (1.5-2)	4 (4-5)	<0.01
8	2 (2-2.5)	4 (3.75-4)	<0.01
12	1 (1-2)	3 (3-4)	<0.01
24	1 (0.5-1)	3 (2-3)	<0.01

*Data are presented as median [IQR] and analyzed by Mann-Whitney U-test. PCB, psoas compartment block; SFIB, supra-inguinal fascia iliaca compartment block*.

**Table 3:** Percentage of patients with a NRS pain score ≤ 3.

**Time (h)**	**PCB (*n* = 29)**	**SFIB (*n* = 30)**	***P***
0	28 (96.6%)	12 (40%)	<0.01
4	28 (96.6%)	5 (16.7%)	<0.01
8	29 (100%)	7 (23.3%)	<0.01
12	29 (100%)	19 (63.3%)	<0.01
24	29 (100%)	28 (93.3%)	<0.01

*Data are presented as number (%) and analyzed by Chi-square test. PCB, psoas compartment block; SFIB, supra-inguinal fascia iliaca compartment block*.

Postoperatively, only 13.8% of patients in the PCB group received administration of sufentanil, in contrast to 63.3% in the SFIB group (*P* < 0.01, Table 4). Sufentanil consumption in the first half hour was significantly increased in the SFIB group as compared to the PCB group (0.1 [0-0.2] vs. 0 [0-0] mcg/kg, *P* < 0.01). In addition, the patients in the PCB group had lower demands for PCA compared with the SFIB group within 24 h after extubation (2 [1-2] vs. 5 [3-6], *P* < 0.01, Table 5).

**Table 4:** Postoperative sufentanil consumption and administration.

	**PCB (*n* = 29)**	**SFIB (*n* = 30)**	***P***
Sufentanil consumption (mcg/kg)	0 (0-0)	0.1 (0-0.2)	<0.01^*^
Sufentanil administration (%)	4 (13.8%)	19 (63.3%)	<0.01^**^

Continuous data are presented as median [IQR] and categorical data are presented as number (%), and were analyzed by ^*^ Mann-Whitney U-test and ^**^ Chi-square test, respectively. PCB, psoas compartment block; SFIB, supra-inguinal fascia iliaca compartment block.

**Table 5:** PCA demands within the first 24 hours after extubation.

	**PCB group (*n* = 29)**	**SFIB group (*n* = 30)**	***P***
PCA demands	2 (1-2)	5 (3-6)	<0.01

*Data are presented as number (%) and analyzed by Mann-Whitney U-test. PCA, patient-controlled analgesia; PCB, psoas compartment block; SFIB, supra-inguinal fascia iliaca compartment block*.

## Discussion

In pediatric DDH patients with late presentation or failure of non-surgical treatment, surgical management may be the only option and indicated. For children with complex deformity or children aged > 3 years, closed reduction and spica casting are not preferred and both acetabuloplasty and femoral osteotomy are commonly required to stabilize an open reduction (13). After skin incision, the surgeon performs acetabuloplasty combined with femoral osteotomy and dissection of surrounding muscles. This surgical operation may result in moderate-to-severe pain, which is an important risk factor for postoperative recovery of the hip structure and function. The femoral and obturator nerves and some branches of sciatic nerve innervate the surgical area, thus a satisfactory analgesic effect could be achieved by successful block of these peripheral nerves theoretically (14–16).

With the development of anesthesia techniques, multimodal analgesia has become a new strategy for perioperative pain control in the hip surgeries. Regional anesthesia plays a crucial role in a multimodal regimen and has been shown to reduce systemic narcotic consumption and the related side effects (17). A previous study of total hip arthroplasty in adult populations found that patients receiving PCB reported a similar satisfaction with pain relief as compared to who having fascia iliaca block (18). Nevertheless, the pediatric patients with the DDH surgery undergoing PCB preoperatively demonstrated a better analgesic satisfaction, compared with who receiving SFIB in this study.

The acute pain of hip surgeries might occur within 24 h after operation, thus main outcomes were observed within this period. The patients in the PCB group had significantly lower NRS pain scores compared to the SFIB group after operation at all time points within the initial 24 h, especially within 12 h. And the percentages of the patients with a NRS score ≤ 3 at all time points in the PCB group were significantly higher than those in the SFIB group. The results demonstrated that the children in the PCB group only experienced a mild pain and gained a superior pain relief, on the contrary, the children in the SFIB group had a more severe pain.

In the present study, this difference was underlined by the significantly lower need to integrate the systemic analgesia with sufentanil in the PCB cohort compared to the SFIB cohort. Meanwhile, frequency of administration of analgesics through PCA in the PCB group was lower than that in the SFIB group at all time points within the initial 24 h. These results also indicated that the patients in the PCB group obtained relatively greater pain relief when compared with the SFIB group in terms to the analgesic efficacy. Though intravenous analgesia is simple to operate and has definite effects, the pain control is not as good as epidural analgesia and peripheral nerve block. And opioids are related to an increased risk of delirium, and the side effects such as hypotension, respiratory depression, constipation and confusion (19). Regional nerve block combined with intravenous analgesia contributes to reducing the need for opioid drops and the related adverse effects (20). In the current study, it was demonstrated that PCB or SFIB combined with intravenous PCA provided a great benefit for acute moderate-to-severe postoperative pain and reduced the sufentanil consumption in the pediatric DDH surgeries, furthermore, PCB worked better.

In the PCB group, local anesthetics could greatly block the transmission of surgical pain stimulation and weaken postoperative neuron sensitization owing to stronger effective diffusion in the lumbar plexus region compared to the SFIB group. An incomplete lumbar plexus block was observed in the SFIB group irrespective of easier spread of local anesthetics in children, as has been reported in the adult patients undergoing the hip surgery (11). The femoral nerve runs behind the iliac fascia with the lateral femoral cutaneous nerve, the obturator nerve and the genital femoral nerve. The previous studies showed that the block success rate of PCB on these nerves was significantly better than fascia iliaca block (21, 22). The results also indicated the onset of PCB was fast and it took about 20 min to achieve the ideal anesthetic effect. Conversely, the complete block rate of fascia iliaca block after 4 h was not changed and the main reason was the low success rate of fascia iliaca block on the obturator nerve.

A few studies have reported that PCB is associated with an increasing risk of complications such as seizures, and retroperitoneal hematoma, and spinal or epidural diffusion of the injected local anesthetics (12). Using ultrasound with blocks, a decline was revealed in nerve damage and vessel injury. It is recommended to use ultrasound during peripheral block interventions to prevent these possible adverse impacts (23). No cases were reported with nerve block-related complications in either group. There were no significant differences in postoperative hospital stay between groups in this study. SFIB took significantly shorter time to achieve its blocking operation than PCB due to its simplicity and the good acceptance by patients. In addition, several studies have demonstrated that the lateral femoral cutaneous nerve could be blocked sufficiently by SFIB in adults (24–26). And there needs further work to evaluate the value of SFIB in child patients who will undergo anterior femoral surgery.

The recommended dose of ropivacaine in pediatric nerve blocks in the Miller's Anesthesia was followed for SFIB and PCB in this study, that is, no more than 3 mg/kg. Besides, the bolus injection of local anesthetics was administered into the interstitial space for both SFIB and PCB. European and American Societies of Regional Anesthesia (ESRA-ASRA) recommended doses for a sciatic nerve block is 0.5-1.5 mg/kg (27). Based on the aforementioned, 0.25% ropivacaine and a total dose of 1 ml/kg in SFIB and PCB were applied, whereas the lowest recommended dose of 0.5 mg/kg in sciatic nerve block was used. The required dose ratio of SFIB/PCB to sciatic nerve block was 8:2.

A major limitation of this study was that dermatome sensation testing was not performed for the reason that all patients were still under general anesthesia after nerve block. This could affect the estimation of the duration and effective site of the nerve block. And NRS pain scores may not fully represent patient pain perception.

## Conclusion

This study demonstrated that PCB supplied a better perioperative pain management in the pediatric patients with the DDH surgeries compared to SFIB. These results were supported by the significantly fewer opioid supplements and PCA demands administered to the patients who received PCB, along with the lower NRS pain scores. For this reason. we are of the opinion that PCB can be a great option in the pediatric DDH surgeries. But there is a need for further studies so that PCB can be affirmed as the more ideal technique.

## Data Availability Statement

The original contributions presented in the study are included in the article/supplementary material, further inquiries can be directed to the corresponding author/s.

## Ethics Statement

The studies involving human participants were reviewed and approved by the Institutional Review Board of Children's Hospital of Chongqing Medical University. Written informed consent to participate in this study was provided by the participants' legal guardian/next of kin.

## Author Contributions

JQ and KC: writing and data analysis. SYu: study design, data review, and article final review. SYa: data analysis. YC: article review. JQ and YC: data collection. All authors contributed to the article and approved the submitted version.

## Conflict of Interest

The authors declare that the research was conducted in the absence of any commercial or financial relationships that could be construed as a potential conflict of interest.

## Publisher's Note

All claims expressed in this article are solely those of the authors and do not necessarily represent those of their affiliated organizations, or those of the publisher, the editors and the reviewers. Any product that may be evaluated in this article, or claim that may be made by its manufacturer, is not guaranteed or endorsed by the publisher.
